# FASE-CPHG Study: identification of asthma phenotypes in the French Severe Asthma Study using cluster analysis

**DOI:** 10.1186/s12931-021-01723-x

**Published:** 2021-05-04

**Authors:** Chantal Raherison-Semjen, Eric Parrat, Cécilia Nocent-Eijnani, Gilles Mangiapan, Anne Prudhomme, Jean-Philippe Oster, Corinne Aperre de Vecchi, Cyril Maurer, Didier Debieuvre, Laurent Portel, The CPHG (College of French Non-academic Hospitals), Appere De Vecchi, Maurer, Lepoulain Doubliez, Iamandi, Gourcerol, Didi, Decroisette, Bertocchi, Moreau, Barbare, Moncelly, Tannous, Kelkel, Rolland, Jouveshomme, Bernier, Hauss, Ould, Vincent, Van Mossevelde, Gallego, Merzoug, Haddad, Allibe, Guerrero, Jarjour, Haouachi, Goutorbe, Morel, Lemaire, Russier, Roge, Dumont, Cavestri, Just, Colin, Goupil, Mansour, Paris, Philippe, Boitiaux, Simon, Marcq, Bizieux, Guibert, Caby, Lecuyer, Merlusca, Roy, Blanc, Langelot, Tagu, Leveiller, Duriel-Niel, Coëtmeur, Ilie, Raspopa, Courdeau-Labourie, Demaegdt, Lerousseau, Rotomondo, Gramada, Cornu, Petit, Kraemer, Huy, Gentil, Luciani, Lucena, Michaux, Maitre, De Faverges, Fouret, Goarant, Bara, Hamoudi, Maetz, Yousef, Lungoci, Al Freijat, Assemi, Beysens, Clarissou, Karimo, Saadi, Barre, Farny, Romand, Leleu, Bernard, Marangoni, Fevrier, Paillot, Bonnefoy, Manoila, Abraham, Lacroix, Virally

**Affiliations:** 1 Groupe Hospitalier Sud, Hôpital Haut-Lévêque CHU Bordeaux, Pessac, France; 2 INSERM U1219 Université de Bordeaux, Bordeaux, France; 3 Centre Hospitalier de Polynésie Française, Hôpital du Taaone, Papeete, French Polynesia; 4 Centre Hospitalier de la Côte Basque, Bayonne, France; 5 Service de Pneumologie, Centre Hospitalier Intercommunal de Créteil, Créteil, France; 6 Clinique Médicale et Cardiologique d’Aressy, Pau, France; 7 Service de Pneumologie, Centre Hospitalier Louis Pasteur, Colmar, France; 8 Centre Hospitalier d’Argenteuil, Argenteuil, France; 9 GHI Montfermeil, Montfermeil, France; 10 Service de Pneumologie, Groupe Hospitalier de la Région Mulhouse Sud-Alsace, Hôpital Émile Muller, Mulhouse, France; 11 Centre Hospitalier Robert Boulin de Libourne, Site Robert Boulin, Libourne, France

**Keywords:** Severe asthma, Phenotypes, Cluster analysis

## Abstract

**Background:**

In France, data regarding epidemiology and management of severe asthma are scarce. The objective of this study was to describe asthma phenotypes using a cluster analysis in severe asthmatics recruited in a real world setting.

**Methods:**

The study design was prospective, observational and multicentric. The patients included were adults with severe asthma (GINA 4–5) followed-up in French Non Academic Hospital between May 2016 and June 2017. One hundred and seven physicians included 1502 patients. Both sociodemographic and clinical variables were collected. Hierarchical cluster analysis was performed by the Ward method followed by k-means cluster analysis on a population of 1424 patients.

**Results:**

Five clusters were identified: cluster 1 (n = 690, 47%) called early onset allergic asthma (47.5% with asthma before 12 years), cluster 2 (n = 153, 10.5%): obese asthma (63.5% with BMI > 30 kg/m^2^), cluster 3 (n = 299, 20.4%): late-onset asthma with severe obstructive syndrome (89% without atopy), cluster 4 (n = 143, 9.8%): eosinophilic asthma (51.7% had more than 500 eosinophils/mm^3^), and cluster 5 (n = 139, 9.5%): aspirin sensitivity asthma (63% had severe asthma attacks).

**Conclusions:**

In our population of adults with severe asthma followed by pulmonologists, five distinct phenotypes were identified and are quite different from those mentioned in previous studies.

**Supplementary Information:**

The online version contains supplementary material available at 10.1186/s12931-021-01723-x.

## Introduction

Asthma is a heterogeneous disease that presents with a variety of symptoms and variable response to medication.

Management of mild to moderate asthma is based on the same treatment for each patient, variable according to asthma control and exacerbations risk [1] (GINA 2018). By contrast, as patients with difficult-to-treat asthma or severe asthma had a high rate of exacerbations and poor asthma control and poor quality of life despite management, improvement in therapeutic management had leaded to better understanding in asthma phenotypes.

A decade ago, asthma phenotypes were defined by two criteria i.e. atopic status and age onset of asthma (childhood versus adulthood) (Wenzel et al.). Since then, Enfumosa Network [2] identified that patients with chronic severe asthma were more likely to be female, overweighted, less atopic and pointed out exposure to aspirin for some subjects. The SARP consortium in USA identified three clusters in adult patients with severe asthma [3]: early onset allergic asthma, late onset non-atopic asthma, severe asthma with fixed airflow. Then, the TENOR project found some similarities with the SARP clusters, for four of five clusters [4]. Interestingly, associations were made between asthma phenotypes and asthma-related health outcomes i.e. quality of life. In these phenotypes, atopic status but also non-white race were distinguishing variables for both children and adolescents. In Europe, some severe asthma registries have been developed in UK [5], in Belgium [6], and in Italy [7]. According to the Belgium Registry, the majority of severe asthmatics were female and atopic, revealing that description of patients depends on existence of network and inclusion of patients at baseline [6]. The same snapshot was described in the Italian Registry [7]. By contrast, in the UK registry [5], five clusters were described: atopic early onset asthma, obese with late onset asthma, least severe asthma, eosinophilic late onset-asthma and fixed airflow obstruction. They also find a poor stability in this longitudinal analysis.

In France, data regarding severe asthma management are scarce, a recent study estimated that prevalence of severe asthma was about 3.8% [8], in line in estimated prevalence of severe asthma 3.6% in previous surveys [9]. In addition, two thirds of patients with severe asthma are managed in non-specialized environment [10]. 9.8% (range 3.5–17.5%) of patients with severe asthma in real life was found to be eligible for enrolment in the phase III trials [11]. FASE-CPHG (France Asthme Sévère—Collège des Pneumologues des Hôpitaux Généraux) was built in 2016 as descriptive, multicentric and observational cross-sectional study in patients with severe asthma conducted in general hospitals in France.

The aim of our study was to describe the clinical phenotypes of severe asthma adults, in a real-life study in France using cluster analysis.

## Methods

### Study population

CPHG is a collaborative group of pulmonologists working in non-academic hospitals. One hundred and ten centers accepted to participate to the study. The methodology and descriptive analysis have been published elsewhere [12].

This study was approved by the local ethics committee (Comité Consultatif sur le Traitement de l’Information en matière de Recherche dans le domaine de la Santé (CCTIRS)) and was conducted according to the French law and guidelines on epidemiological and descriptive studies.

Pulmonologists from an extensive list of practitioners were contacted to confirm their willingness to participate in the FASE-CPHG observational study. During the inclusion period, selected pulmonologists were required to recruit all patients who meet the eligibility criteria to ensure exhaustivity. Moreover, and for the same reason, patients who refused to participate in the study were logged in a non-inclusion register.

To join the study, patients must have fulfilled all of the following criteria: aged over 18 years old with a severe asthma diagnosis according to the physician and based on Global INitiative for Asthma (GINA) [1]. All subjects were informed during a regular visit by the physician before being enrolled. Patients diagnosed with solid cancer or malignant hemopathy where excluded and also those who refuse to participate in the study.

According to GINA criteria, severe asthma is defined as asthma that requires Step 4 or 5 treatment to prevent it from becoming ‘uncontrolled’, or asthma that remains ‘uncontrolled’ despite this treatment. Uncontrolled step 3 patients were also considered as severe asthmatics as the adjustment strategy in case of uncontrolled asthma per 3 months would be to step up treatment up to step 4. After validation by the physicians, patients only treated with short acting beta agonist were excluded from analyses as it is considered as non-severe asthma according to GINA criteria.

### Patient data collection

Physician completed a secure electronic Case Report Form (eCRF), during a regular visit on patient characteristics (sociodemographic data, potential asthma triggers, medical history, comorbidities, clinical parameters, spirometry, blood eosinophils) and asthma ongoing treatment for all patients seen during the study period.

In addition, patients were required to fill in auto-questionnaires comprising items on asthma control (Asthma Control Test (ACT)), anxiety and depression (Hospital Anxiety and *Depression Scale* (HADS)).

### Data management

Data were entered into databases managed by Kappa Santé, Paris, France. Duplicates were identified with indirectly nominative data (initial, age and sex) and reviewed with participant pulmonologists. In addition of online control present on eCRF, data were reviewed before database was frozen freeze for other errors, omissions or inconsistencies by a scientific committee.

Patients enrolled by participant physician with no completed CRF were removed from the analysis.

### Statistical analysis

All statistical analyses were performed using SAS (version 9.4, SAS Institute Inc., Carey, North Carolina, USA). P value < 0·05 was regarded as statistically significant.

Qualitative variables are summarized as raw and frequencies; number of missing data is specified. Quantitative data is expressed as numbers of analyzed values, mean with standard deviation.

Asthma control was evaluated using the ACT, a 5-item questionnaire (activity limitation, shortness of breath, night symptoms, use of rescue medication and self-perception of asthma control). Each parameter was scored from 1 (poorly control) to 5 (well controlled). The HADS was used to evaluate anxiety and depression symptoms in patients. The HADS is based on 14-items and produces two scales: one for anxiety (HADS-A) and one for depression (HADS-D). A score ≥ 11 on either scale indicate a definitive case whereas score < 7 generally indicates an absence of the trouble.

### Cluster analysis

Eighteen variables (gender, BMI, age, age of asthma onset, severe asthma attacks with ICU, FEV1, clinical atopy, exacerbations, allergenic sensitization, aspirin intolerance, nasal polyposis, chronic rhinitis, apnea syndrome, reflux, hypertension, smoking status, and eosinophils count) have been included in the analysis. Missing data were most frequent for eosinophils count, and two methods have been performed with and without imputation data.

**Fig. 1:**
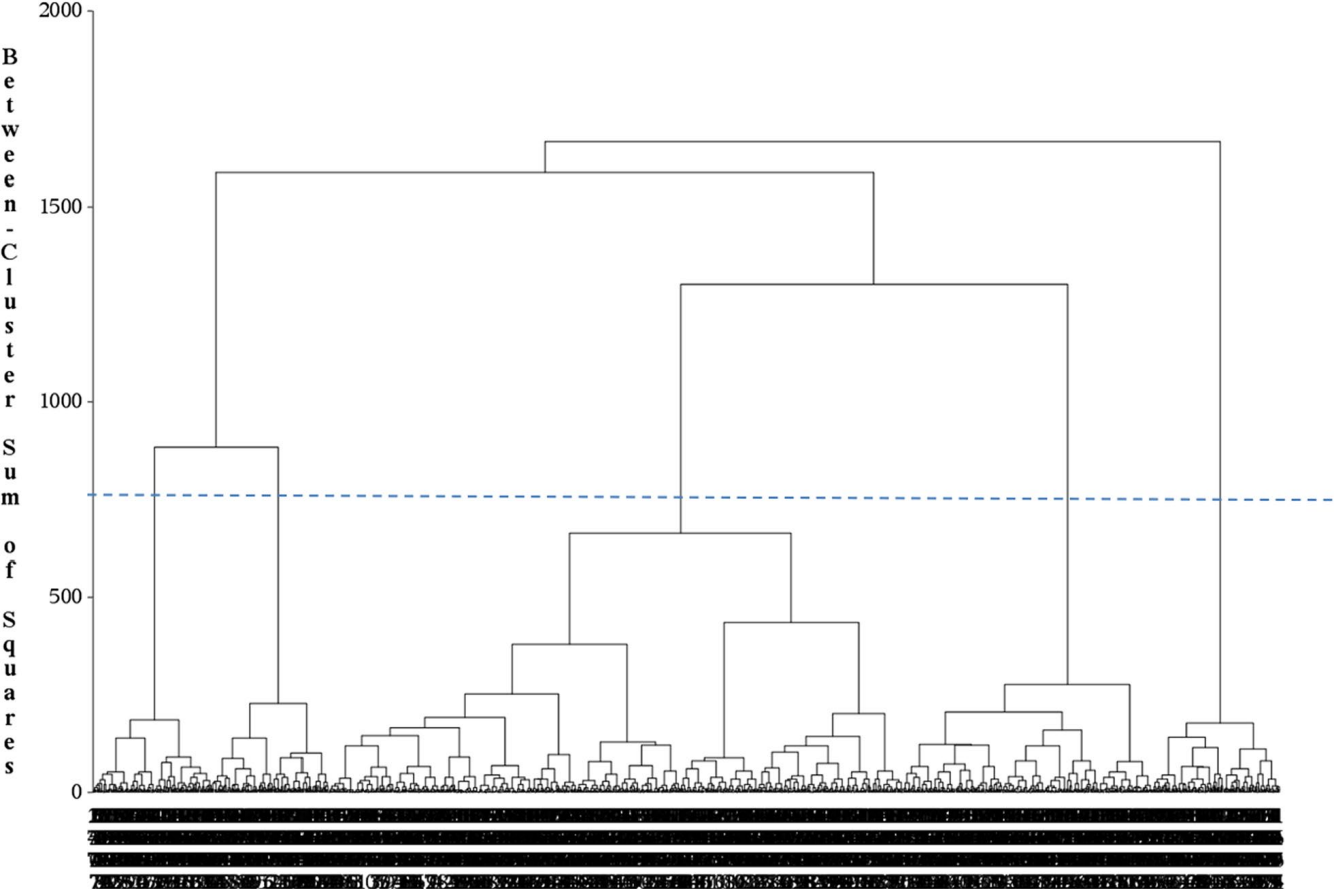
Hierarchical bottom-up classification using Ward Method

A hierarchical bottom-up classification method using Ward's method is then carried out, using an agglomeration (ascending) approach and a ward distance (Fig. 1). With each generation of clusters, samples are merged into larger clusters to minimize the sum of intra-cluster squares, while maximizing the sum of inter-cluster squares. In order to compare the differences between the resulting clusters, ANOVA, the Kruskal–Wallis test and The Pearson Khi Two test are used respectively for continuous parametric variables, continuous non-parametric variables and categorical variables (classes). The dendrograms were produced and were examined to help to determine the number of clusters as shown on Fig. 1.

## Results

One thousand and four hundred twenty four patients from 107 centers were included in this analysis. A five cluster model best described the dataset. Their characteristics are as shown in Table 1.

**Table 1:** Clinical characteristics of FASE-CPHG severe asthma clusters

		Cluster 1 Early onset atopic (N = 690)	Cluster 2 Obese (N = 153)	Cluster 3 Late-onset (N = 299)	Cluster 4 Eosinophilic (N = 143)	Cluster 5 Aspirin-sensitivity (N = 139)	Patients N = 1424	p
Gender	Male	229 (33.2%)	81 (52.9%)	112 (37.5%)	69 (48.3%)	40 (28.8%)	531 (37.3%)	< 0.0001
	Female	461 (66.8%)	72 (47.1%)	187 (62.5%)	74 (51.7%)	99 (71.2%)	893 (62.7%)	
BMI	< 21 kg/m^2^	23 (3.3%)	1 (0.7%)	10 (3.3%)	4 (2.8%)	6 (4.3%)	44 (3.1%)	< 0.0001
	21–25 kg/m^2^	278 (40.3%)	12 (7.8%)	101 (33.8%)	59 (41.3%)	44 (31.7%)	494 (34.7%)	
	25–29 kg/m^2^	198 (28.7%)	43 (28.1%)	119 (39.8%)	48 (33.6%)	57 (41%)	465 (32.7%)	
	Obese > 30 kg/m^2^ Median	191 (27.7%) 26	97 (63.4%) 32.9	69 (23.1%) 26	32 (22.4%) 25.7	32 (23%) 26.3	421 (29.6%) 26.5	
Age at baseline	[18–40]	216 (31.3%)	4 (2.6%)	18 (6%)	18 (12.6%)	20 (14.4%)	276 (19.4%)	< 0.0001
	[40–60]	307 (44.5%)	62 (40.5%)	87 (29.1%)	59 (41.3%)	56 (40.3%)	571 (40.1%)	
	60 years and more Median	167 (24.2%) 50	87 (56.9%) 61	194 (64.9%) 65	66 (46.2%) 59	63 (45.3%) 57.5	577 (40.5%) 56	
Age at onset of asthma	< 12 years	359 (52%)	33 (21.6%)	28 (9.4%)	32 (22.4%)	35 (25.2%)	487 (34.2%)	< 0.0001
	[12–40]	256 (37.1%)	52 (34%)	91 (30.4%)	67 (46.9%)	67 (48.2%)	533 (37.4%)	
	40 years and more Median	75 (10.9%) 12	68 (44.4%) 36	180 (60.2%) 44	44 (30.8%) 30	37 (26.6%) 30	404 (28.4%) 24	
Severe asthma attacks	Ever	362 (52.5%)	93 (60.8%)	135 (45.2%)	60 (42%)	88 (63.3%)	738 (51.8%)	< 0.001
FEV1, % pred	Means (± sd)	73.2 (± 19.3)	68 (± 21)	68.2 (± 22.2)	76.7 (± 20.2)	75.7 (± 22)	< 0.0001	

Cluster 1 comprised 47% of the cohort, had early onset allergic asthma (52% with asthma before 12 years), cluster 2 (n = 153, 10.5%,) comprised obese asthma (63.4% with BMI > 30 kg/m^2^), more often men, cluster 3 (n = 299, 20.4%) had late-onset asthma with 60.2% having asthma after 40 years old, cluster 4 (n = 143, 9.8%) comprised eosinophilic asthma (51.7% had more than 500 eosinophils/mm^3^), and cluster 5 (n = 139, 9.5%) had aspirin sensitivity asthma (with 63% had severe asthma attacks).

Regarding allergenic sensitization, 79.3% of patients in cluster 1 had skin prick test positivity. Nasal polyposis was more reported in patients of cluster 4, in patients having eosinophilic profile, and in cluster 5 in patients having also aspirin sensitivity. Chronic rhinitis and rhinosinusitis were more frequent in cluster 4 and 5. By contrast, obstructive apnea syndrome was exclusively reported in cluster 2, with others comorbidities as reflux, hypertension and smoking history (Table 2).

**Table 2:** Comorbidities of FASE-CPHG severe asthma clusters

		Cluster 1 Early onset atopic (N = 690)	Cluster 2 Obese (N = 153)	Cluster 3 Late-onset (N = 299)	Cluster 4 Eosinophilic (N = 143)	Cluster 5 Aspirin-sensitivity (N = 139)	Patients N = 1424	p
Allergenic sensitization, n = 1158	SPT positivity	547 (79.3%)	81 (52.9%)	33 (11%)	67 (46.9%)	83 (59.7%)	811 (70%)	< 0.0001
Aspirin sensitivity	Yes	1 (0.1%)	12 (7.8%)	0 (0%)	1 (0.7%)	139 (100%)	153 (10.7%)	< 0.0001
Nasal polyposis	Yes	12 (1.7%)	21 (13.7%)	5 (1.7%)	143 (100%)	77 (55.4%)	258 (18.1%)	< 0.0001
Allergic rhinitis	Yes	323 (46.8%)	69 (45.1%)	96 (32.1%)	80 (55.9%)	74 (53.2%)	642 (45.21%)	< 0.0001
Rhinosinusitis	Yes	137 (19.9%)	33 (21.6%)	55 (18.4%)	65 (45.5%)	62 (44.6%)	352 (24.7%)	< 0.0001
Obstructive apnea syndrome	Yes	0 (0%)	153 (100%)	1 (0.3%)	1 (0.7%)	1 (0.7%)	156 (11%)	< 0.0001
Reflux history	Yes	231 (33.5%)	75 (49%)	129 (43.1%)	50 (35%)	70 (50.4%)	555 (39%)	< 0.0001
Hypertension	Yes	119 (17.2%)	83 (54.2%)	102 (34.1%)	20 (14%)	37 (26.6%)	361 (25.4%)	< 0.0001
Smoking history	Active smoker	108 (15.7%)	8 (5.2%)	32 (10.7%)	7 (4.9%)	11 (7.9%)	166 (11.7%)	< 0.0001
	Ex-smoker	176 (25.5%)	61 (39.9%)	85 (28.4%)	49 (34.3%)	40 (28.8%)	411 (28.9%)	
	No-smoker	406 (58.8%)	84 (54.9%)	182 (60.9%)	87 (60.8%)	88 (63.3%)	847 (59.5%)	
Blood eosinophil count	< 300	331 (48%)	66 (43.1%)	150 (50.2%)	36 (25.2%)	63 (45.3%)	646 (45.4%)	< 0.0001
	[300–500]	176 (25.5%)	46 (30.1%)	63 (21.1%)	33 (23.1%)	28 (20.1%)	346 (24.3%)	
	≥ 500	183 (26.5%)	41 (26.8%)	86 (28.8%)	74 (51.7%)	48 (34.5%)	432 (30.3%)	

Food allergy and drug allergy was more associated in cluster 5, most of the comorbidities were associated with cluster 2 i.e. diabetes, ischemic cardiopathy, and depression (Table 3).

**Table 3:** Comorbidities of FASE-CPHG severe asthma clusters

		Cluster 1 Early onset atopic (N = 690)	Cluster 2 Obese (N = 153)	Cluster 3 Late-onset (N = 299)	Cluster 4 Eosinophilic (N = 143)	Cluster 5 Aspirin-sensitivity (N = 139)	Patients analysed n = 1424	p
Fernand widal syndrome	Yes	3 (0.4%)	6 (3.9%)	0 (0%)	16 (11.2%)	72 (51.8%)	97 (6.8%)	< 0.0001
Food allergy	Yes	78 (11.3%)	17 (11.1%)	12 (4%)	14 (9.8%)	25 (18%)	146 (10.3%)	< 0.01
Drug allergy	Yes	69 (10%)	28 (18.3%)	35 (11.7%)	15 (10.5%)	54 (38.8%)	201 (14.1%)	< 0.0001
Atopic dermatitis	Yes	130 (18.8%)	18 (11.8%)	19 (6.4%)	19 (13.3%)	33 (23.7%)	219 (15.4%)	< 0.0001
Allergic broncho-pulmonary aspergillosis	Yes	22 (3.2%)	2 (1.3%)	6 (2%)	1 (0.7%)	5 (3.6%)	36 (2.5%)	0.28
Vascularitis	Yes	6 (0.9%)	1 (0.7%)	5 (1.7%)	3 (2.1%)	0 (0%)	15 (1.1%)	0.33
Diabetus	Yes	46 (6.7%)	35 (23%)	42 (14.1%)	7 (4.9%)	11 (8%)	141 (10%)	< 0.0001
Ischemic cardiopathy	Yes	22 (3.2%)	16 (10.5%)	17 (5.7%)	9 (6.3%)	6 (4.3%)	70 (4.9%)	< 0.001
Osteoporosis	Yes	58 (8.4%)	17 (11.1%)	36 (12.1%)	23 (16.1%)	19 (13.7%)	153 (10.8%)	0.16
Anxiety (HAD-A)	Means (Sd)	7.6 (± 4.5)	7.5 (± 4.5)	7.3 (± 4.2)	7 (± 4.4)	7.1 (± 4.7)	7.4 (± 4.4)	0.52
Depression (HAD-D)	Means (Sd)	4.6 (± 3.6)	5.6 (± 4.1)	5.4 (± 3.9)	5.3 (± 4.2)	5.3 (± 3.7)	5 (± 3.8)	< 0.01
Number of comorbidities (outside ENT)	0	247 (35.8%)	0 (0%)	57 (19.1%)	47 (32.9%)	27 (19.4%)	378 (26.6%)	
	1	212 (30.8%)	24 (15.7%)	92 (30.9%)	52 (36.4%)	42 (30.2%)	422 (29.7%)	
	2	120 (17.4%)	33 (21.6%)	75 (25.2%)	23 (16.1%)	33 (23.7%)	284 (20%)	
	3 or more	110 (16%)	96 (62.7%)	74 (24.8%)	21 (14.7%)	37 (26.6%)	338 (23.8%)	

Osteoporosis was not associated with specific cluster. The number of comorbidities was particularly high in cluster 2. The frequency of frequent exacerbation profile was present in each cluster, however the number of exacerbations requiring an increase of treatment either oral corticosteroids or inhaled treatment was higher in eosinophilic cluster (named cluster 4) and aspirin sensitivity (cluster 5). Patients from cluster 2 had higher emergency visits compared to others patients. Absenteeism related to asthma was more frequent in patients from cluster 1 and cluster 5.

House dust mite sensitization was more related in cluster 1 having early onset asthma, and was very low in late-onset asthma (cluster 3). Sensitization to molds and cockroaches were also lower in late-onset asthma cluster, non-atopic. The distribution of blood eosinophils is presented by cluster (Table 3). Despite a large proportion of patients having more than 500 eosinophil counts in cluster 4, eosinophilic distribution was heterogeneous across the different clusters. Among 1462 patients, 19% had missing data regarding blood eosinophils or IgE level, 55.6% (n = 814) (Additional file 1: Table S1) had blood eosinophils count and IgE level, 19% had blood eosinophils count but no IgE, and 6.4% had IgE levels but no blood eosinophils count available. Finally, 12.7% of the patients had low TH2 profile, 13% eosinophilic non-allergic profile, 28.5% had allergic non-eosinophilic profile and 26.9% had eosinophilic and allergic profile. Lung function results showed that the high proportion of patients having FEV1 < 60% was in cluster 2 (obese patients with comorbidities) and in cluster 3 late-onset non-atopic asthma Additional file 1: Table S2). Obstructive syndrome with low FEV1/FVC ratio was more important in cluster 2 and cluster 5 (aspirin sensitivity cluster). Obstruction of small airways was common whatever the cluster group (Table 4).

**Table 4:** History of exacerbations in FASE-CPHG severe asthma clusters

		Cluster 1 Early onset atopic (N = 690)	Cluster 2 Obese (N = 153)	Cluster 3 Late-onset (N = 299)	Cluster 4 Eosinophilic (N = 143)	Cluster 5 Aspirin-sensitivity (N = 139)	Patients analysed n = 1424	p
More than 2 exacerbations	Yes	442 (64.1%)	101 (66%)	185 (61.9%)	97 (67.8%)	100 (71.9%)	925(65%)	0.26
Number of exacerbations with increased of OCS or ICS	Means (± sd)	2.6 (± 3.2)	2.6 (± 3)	2.2 (± 2.6)	2.9 (± 3.6)	2.9 (± 3.1)	2.5 (± 3.1)	0.03
	Median	2	2	1	2	2	2	
	Min–Max	0–20	0–16	0–15	0–20	0–20	0–20	
Number of hospitalizations during the 12 past months	Means (± sd)	0.4 (± 1.2)	1 (± 1.8)	0.6 (± 1.3)	0.4 (± 1.5)	0.4 (± 1)	0.5 (± 1.3)	< 0.0001
	Min–Max	0–12	0–12	0–12	0–15	0–6	0–15	
Number of emergency visits during the 12 past months	Means (± sd)	0.6 (± 1.7)	0.9 (± 2.1)	0.6 (± 1.5)	0.5 (± 1.8)	0.5 (± 1.1)	0.6 (± 1.7)	0.04
	Min–Max	0–20	0–15	0–12	0–17	0–6	0–20	
Number of medical visits during the 12 past months	Means (± sd)	2.6 (± 3.1)	2.8 (± 3.5)	2.2 (± 2.5)	3.1 (± 4)	2.9 (± 3.2)	2.6 (± 3.1)	0.19
	Median	2	2	2	2	2	2	
	Min–Max	0–20	0–20	0–12	0–20	0–20	0–20	
Absenteism related to asthma during the 12 past months	Yes	113 (16.4%)	15 (9.8%)	24 (8%)	16 (11.2%)	21 (15.1%)	189 (13.3%))	< 0.0001

Regarding therapeutic management (Additional file 1: Table S3), a proportion of patients was still not-compliant to treatment according to Moriski scale (< 3). Anti-leukotrienes were more prescribed than anti-cholinergic treatment. The prescription of regular oral corticosteroids was higher in cluster 2 (obese patient with comorbidities). Omalizumab was prescribed in one third of the patients. A high proportion of patients had no physical activity, the greatest proportion was belong to cluster 2 (obese patients with comorbidities) (Table 5).

**Table 5:** Allergenic sensitization (SPT positivity or Specific IgE) and blood eosinophilic count

		Cluster 1 Early onset atopic (N = 690)		Cluster 2 Obese (N = 153)		Cluster 3 Late-onset (N = 299)	Cluster 4 Eosinophilic (N = 143)	Cluster 5 Aspirin-sensitivity (N = 139)	Patients with allergic tests N = 1158	p
Allergic sensitization	Yes	547 (91%)		81 (65.9%)		33 (16.9%)	67 (56.8%)	83 (68.6%)	811 (70%)	< 0.0001
House dust mite	Yes	432 (71.9%)		66 (53.7%)		17 (8.7%)	49 (41.5%)	66 (54.5%)	630 (54.4%)	< 0.0001
Pollens	Yes	317 (52.7%)		50 (40.7%)		13 (6.7%)	31 (26.3%)	52 (43%)	463 (40%)	< 0.0001
Pets	Yes	252 (41.9%)		34 (27.6%)		7 (3.6%)	25 (21.2%)	41 (33.9%)	359 (31%)	< 0.0001
Molds	Yes	98 (16.3%)		14 (11.4%)		7 (3.6%)	21 (17.8%)	19 (15.7%)	159(13.7%)	< 0.001
Cockroaches	Yes	37 (6.2%)		6 (4.9%)		2 (1%)	6 (5.1%)	4 (3.3%)	55 (4.7%)	0.06
Blood eosinophil count (n = 1075)	Means (Sd)	376.7 (± 354.2)		456.6 (± 613.9)		407.4 (± 425.5)	706.9 (± 691)	447.6 (± 399.4)	436 (± 465.6)	< 0.0001
	Min–Max	0–3700		0–3940		0–2340	0–5000	0–2200	0–5000	< 0.0001
Distribution of blood eosinophil count	< 150	112 (22.1%)		23 (18.7%)		66 (30.8%)	12 (10.1%)	23 (20.4%)	236 (22%)	< 0.0001
	[150–300]	142 (28.1%)		33 (26.8%)		48 (22.4%)	16 (13.4%)	28 (24.8%)	267 (24.8%)	
	[300–500]	133 (26.3%)		35 (28.5%)		36 (16.8%)	26 (21.8%)	21 (18.6%)	251 (23.3%)	
	[500–1000]	94 (18.6%)		23 (18.7%)		46 (21.5%)	39 (32.8%)	32 (28.3%)	234 (21.8%)	
	[1000–1500]	18 (3.6%)		2 (1.6%)		11 (5.1%)	20 (16.8%)	4 (3.5%)	55 (5.1%)	
	> = 1500	7 (1.4%)		7 (5.7%)		7 (3.3%)	6 (5%)	5 (4.4%)		

## Discussion

In this large real-life study including difficult-to severe asthmatic patients followed by pulmonologist in non-academic general hospitals, we described five phenotypes of patients using cluster analysis. The five cluster analysis were described cluster 1 (47%) the most atopic with early-onset disease, cluster 2 (10.5%) obese asthmatic with high prevalence of comorbidities (more than 3) including obstructive apnea syndrome, cluster 3 (20.4%) the late-onset asthma without atopy, cluster 4 (9.8%) eosinophilic asthma with nasal polyposis, and cluster 5 with aspirin sensitivity asthma.

Regarding the general characteristics of the population, our population is in line with what had been recently published by the International Severe Asthma Registry [13] and the ERS severe asthma registries [14]. Patients were predominantly female, with overweight or obesity, and non-smoker. Most of patients having uncontrolled asthma on GINA step 5 or on GINA step 4. 65.8% developed also asthma after 12 years old in our population compared to 77.5% in the ISAR registry. The mean number of exacerbation was higher in our population (2.5) vs (1.7) in the ISAR registry, with a significant heterogeneity across countries. Lung function before and after bronchodilator was quite similar to the ISAR global value, with little improvement after bronchodilator. Unfortunately we couldn’t compare the FeNO measurements, as in our study most of practitioners could not access to this evaluation tool. In our population, 47.6% of patients had IgE lower than 200 UI/l compared to half of the ISAR registry, who had lower IgE concentration. In the same trend, 50% of our patients had a blood eosinophils count > 0.3 * 10^9^ cells/L. Allergic rhinitis was the predominant comorbidity, followed by reflux and hypertension. The prevalence of nasal polyps was higher in our population (18%) vs 7.3% in the ISAR registry. We could not compare the prevalence of OSA or cardiovascular comorbidity, or osteoporosis, not reported in the ISAR publication. 16.9% of the patient received regular oral corticosteroids compared to 30.1% of the ISAR cohort. In the ISAR registry, 25.4% of the patients were on biologics, very similar to what we found in our population; however anti-IL5 was not available in France at the beginning of our study, explaining that anti-IgE was the most predominant prescription. Only 5% of patients had azithromycin prescription, lower than 9.2% of the patients from the ISAR registry.

Our cluster analysis revealed 5 clusters, 4 of them have been mostly described in previous studies: the classic early onset severe allergic asthma, the obese asthma with high impairment, the late-onset non-atopic cluster and the eosinophilic phenotype. Enfumosa Network [2] identified previously that patients with chronic severe asthma were more female, more in overweight, less atopic and pointed out exposure to aspirin for some subjects. The SARP consortium in the US identified three clusters in adult patients with severe asthma [3] using unsupervised cluster analyses: early onset allergic asthma, late onset non-atopic asthma, severe asthma with chronic airflow obstruction.Then, the TENOR project found some similarities with the SARP clusters, for four of five clusters [4] using hierarchical clustering. As us, they also identified a phenotype of adult-onset asthma with aspirin sensitivity, which is underreported in the literature. In the UK registry, five clusters [5] have been identified using a two way cluster/mixture analysis with the Bayesian information criterion: atopic early onset asthma, obese with late onset asthma, least severe asthma, eosinophilic late onset-asthma and fixed airflow obstruction. Amelink et al. [15] identified two clusters using K-means nonhierarchical cluster analysis: one with severe eosinophilic inflammation and another with frequent symptoms, high healthcare utilization and low sputum eosinophils. Newby et al. (2014) [16] identified also four clusters: early onset atopic, late-onset in obese patients, eosinophilic asthma, non-atopic with normal lung function and one group with reversible obstruction. The obese asthma phenotype has been already described, however in our analysis, we pointed out that this group was at higher risk of comorbidities, cardiovascular diseases, OSA, ex-smoker status; in addition, two third of them had more than 3 comorbidities outside ENT comorbidities. In addition, 30% of them had prescription of oral corticosteroids, so we cannot formally exclude that this group of patients had comorbidities induced by oral steroids [17]. They also had the worse lung function regarding FEV1 or FEV1/FVC ratio before and after bronchodilatator, and the lowest physical activity compared to the other clusters. One major difference from the other studies was that in our obese cluster, atopy was present in half of the group. The UK registry [16] identified five clusters: atopic early onset asthma, obese with late onset asthma, least severe asthma, eosinophilic late onset-asthma and fixed airflow obstruction. In the eosinophilic phenotype, 20% had nasal polyps whereas in our cluster analysis 100% of the patients had nasal polyps. However, the mean of blood eosinophilic count was very similar. We described also the late-onset non-atopic asthma, which was described previously in Tenor cohort, the main difference with the Sharp study was obesity [4].

Our study had some limitations. The recruitment of severe asthmatic patients was made by pulmonologist from non-academic hospitals, not from university hospitals or primary care, so we cannot generalize the findings of our study to the whole population of severe asthmatics in France. This explained also why FeNO was not available for the majority of the centers. It is noteworthy that FeNO is not a recognized tool for monitoring asthma by the national insurance French authority at the time this paper was written. The SHARP ERS consortium found that severe asthmatic patients in Europe is heterogeneous, and differs in both clinical characteristics and treatment, most of registries enrolled patients being treated in a tertiary care center, however small centers included patients with severe asthma from primary care and second care hospitals [14].

Our statistical analysis had performed imputation algorithms to allow missing values, particularly for eosinophils counts. We made sensitivity analysis with and without imputation, the results did not change. We presented an unbiased statistical cluster analysis technique, which selects the number of the cluster based on the data. However, we must admit that bias could come from the choice of the variables included in the cluster analysis. The confidence that we have in our analysis, is that some of our clusters had been already shown in others registries, in UK or in USA. Another limit of our study is the cross-sectional design of the analysis, so we would not able to ensure stability of the clusters. In addition, there is a heterogeneity in cluster analysis (supervised vs unbiased) in the different studies that made difficult the comparison. None analysis has shown a superiority to another. The impact of OCS on blood eosinophils count is difficult to analyze, patients with OCS seem to have more eosinophils count than patients without OCS, suggesting that patients with OCS could have TH2 profile, compared to the others Additional file 1: Fgure S4, S5, Table S6.

## Conclusion

Despite these limitations, we were able to describe five clusters in a very large population of difficult-to severe asthmatic managed by pulmonologist in non-academic hospitals in France; early onset atopic cluster, obese-late onset asthma with high comorbidities, late-onset non-atopic asthma, eosinophilic asthma with nasal polyps and aspirin sensitivity. Understanding heterogeneity of severe asthma in real life remains an important challenge to input personalized medicine, many of these patients are still excluding for the moment from randomized clinical trials.

## Supplementary Information

**Additional file 1: Table S1.** Blood eosinophils count and IgE levels in FASE severe asthma patients. **Table S2.** Spirometric profiles and FASE-CPHG severe asthma clusters. **Table S3.** Therapeutic management and FASE-CPHG severe asthma clusters. **Figure S4**. Distribution of vems by cluster. **Figure S5**. Distribution of eosinophils by cluster. **Table S6**. Blood count eosinophils according to OCS

## Data Availability

The database is available upon request at Kappa Santé a contract research organization.

## Abbreviations

GINA: Global initiative for asthma
BMI: Body mass index
ACT: Asthma control test
HADS: Hospital anxiety and depression scale
CRF: Case report form
ICU: Intensive Care Unit
FEV1: Flow expiratory volume
IgE: Immunoglobulin E
FEV1/FVC ratio: Flow expiratory volume/forced vital capacity
OCS: Oral corticosteroids
ICS: Inhaled corticosteroids
ENT: Ear-nose-throat
OSA: Obstructive syndrome apnea
FeNO: Exhaled fraction NO
