# Supplementary material for: FASE-CPHG Study: identification of asthma phenotypes in the French Severe Asthma Study using cluster analysis
Source: Respir Res. 2021 May 4;22:136. doi: 10.1186/s12931-021-01723-x (PMC8097842; doi:10.1186/s12931-021-01723-x)
Supplement: Supplementary file 1 — Additional file 1: Table S1. Blood eosinophils count and IgE levels in FASE severe asthma patients. Table S2. Spirometric profiles and FASE-CPHG severe asthma clusters. Table S3. Therapeutic management and FASE-CPHG severe asthma clusters. Figure S4. Distribution of vems by cluster. Figure S5. Distribution of eosinophils by cluster. Table S6. Blood count eosinophils according to OCS [file 12931_2021_1723_MOESM1_ESM.docx]

**Additional information**

**S1 Table: Blood eosinophils count and IgE levels in FASE severe asthma patients**

|  | | Eosinophils count | | |  |
| --- | --- | --- | --- | --- | --- |
|  |  | **<150** | **[150-300[** | **>= 300** | **Patients with both IgE et eosinophils** |
|  |  | **(N=182)** | **(N=203)** | **(N=429)** | **(N=814)** |
| Total IgE (UI/L) | 0-30 | 27 (14.8%) | 19 (9.4%) | 36 (8.4%) | 82 (10.1%) |
|  | 30-100 | 50 (27.5%) | 50 (24.6%) | 60 (14%) | 160 (19.7%) |
|  | 100-200 | 35 (19.2%) | 40 (19.7%) | 68 (15.9%) | 143 (17.6%) |
|  | 200-300 | 16 (8.8%) | 26 (12.8%) | 44 (10.3%) | 86 (10.6%) |
|  | 300-400 | 13 (7.1%) | 7 (3.4%) | 43 (10%) | 63 (7.7%) |
|  | 400-500 | 8 (4.4%) | 12 (5.9%) | 29 (6.8%) | 49 (6%) |
|  | 500-600 | 6 (3.3%) | 5 (2.5%) | 29 (6.8%) | 40 (4.9%) |
|  | 600-700 | 1 (0.5%) | 4 (2%) | 19 (4.4%) | 24 (2.9%) |
|  | 700-800 | 2 (1.1%) | 8 (3.9%) | 10 (2.3%) | 20 (2.5%) |
|  | 800-900 | 3 (1.6%) | 5 (2.5%) | 7 (1.6%) | 15 (1.8%) |
|  | 900-1000 | 1 (0.5%) | 3 (1.5%) | 12 (2.8%) | 16 (2%) |
|  | 1000-1100 | 0 (0%) | 2 (1%) | 5 (1.2%) | 7 (0.9%) |
|  | 1100-1200 | 1 (0.5%) | 3 (1.5%) | 6 (1.4%) | 10 (1.2%) |
|  | 1200-1300 | 2 (1.1%) | 2 (1%) | 9 (2.1%) | 13 (1.6%) |
|  | 1300-1400 | 4 (2.2%) | 1 (0.5%) | 4 (0.9%) | 9 (1.1%) |
|  | 1400-1500 | 0 (0%) | 1 (0.5%) | 6 (1.4%) | 7 (0.9%) |
|  | >1500 | 13 (7.1%) | 15 (7.4%) | 42 (9.8%) | 70 (8.6%) |

S2 Table. Spirometric profiles and FASE-CPHG severe asthma clusters

|  | | **Cluster 1**  **Early onset atopic  (N=690)** | **Cluster 2**  **Obese  (N=153)** | **Cluster 3**  **Late-onset**  **(N=299)** | **Cluster 4**  **Eosinophilic  (N=143)** | **Cluster 5**  **Aspirin-sensitivity  (N=139)** | **p** |
| --- | --- | --- | --- | --- | --- | --- | --- |
| **FEV1 % pred** | means (±sd) | 73.2 (± 19.3) | 68 (± 21) | 68.2 (± 22.2) | 76.7 (± 20.2) | 75.7 (± 22) | <0.0001 |
| **FEV1 group** | < 60% | 153 (24.1%) | 48 (35%) | 101 (36.3%) | 24 (19.2%) | 37 (28.2%) | <0.001 |
|  | [60%-80%[ | 240 (37.8%) | 48 (35%) | 93 (33.5%) | 47 (37.6%) | 36 (27.5%) |  |
|  | >= 80% | 242 (38.1%) | 41 (29.9%) | 84 (30.2%) | 54 (43.2%) | 58 (44.3%) |  |
| **FEV1 % pred after SABA** | means (±sd) | 77.7 (± 19.3) | 72.9 (± 20.3) | 73.4 (± 22.5) | 81.5 (± 18.3) | 81.3 (± 21.7) | <0.0001 |
| **FEV1/FVC ratio** | means (±sd) | 70.2 (± 14) | 71.2 (± 14.9) | 68.8 (± 15.3) | 72.7 (± 13.7) | 69.7 (± 14.2) | <0.0001 |
| **FEF25-75% pred** | means (±sd) | 45 (± 23.8) | 44.4 (± 27.3) | 40.9 (± 23.9) | 50.1 (± 28.4) | 45 (± 24.6) | <0.0001 |

S3 Table. Therapeutic management and FASE-CPHG severe asthma clusters

|  | | **Cluster 1**  **Early onset atopic  (N=690)** | **Cluster 2**  **Obese  (N=153)** | **Cluster 3**  **Late-onset**  **(N=299)** | **Cluster 4**  **Eosinophilic  (N=143)** | **Cluster 5**  **Aspirin-sensitivity  (N=139)** | **p** |
| --- | --- | --- | --- | --- | --- | --- | --- |
| **Adherence (score de Morisky >= 3)** | Yes | 487 (78.7%) | 120 (87.6%) | 220 (85.3%) | 106 (84.8%) | 105 (88.2%) | <0.01 |
| **Inhaled treatment** | ICS | 59 (8.6%) | 12 (7.8%) | 35 (11.7%) | 12 (8.4%) | 15 (10.8%) | 0.5 |
|  | LABA | 49 (7.1%) | 7 (4.6%) | 28 (9.4%) | 11 (7.7%) | 8 (5.8%) | 0.39 |
|  | Fixed combination | 637 (92.3%) | 142 (92.8%) | 259 (86.6%) | 133 (93%) | 130 (93.5%) | 0.03 |
|  | Anticholinergic | 217 (31.4%) | 65 (42.5%) | 118 (39.5%) | 38 (26.6%) | 45 (32.4%) | <0.01 |
| **Anti-leukotriens** | Yes | 373 (54.1%) | 82 (53.6%) | 120 (40.4%) | 84 (58.7%) | 85 (61.2%) | <0.0001 |
| **Oral corticosteroids long –term use** | Yes | 77 (11.2%) | 46 (30.1%) | 62 (20.9%) | 24 (16.8%) | 31 (22.3%) | <0.0001 |
| **Any Biotherapy** | Yes | 198 (28.7%) | 48 (31.4%) | 62 (20.9%) | 49 (34.3%) | 41 (29.5%) | 0.02 |
| **Anti-IgE (omalizumab)** | Yes | 193 (28%) | 46 (30.1%) | 57 (19.2%) | 47 (32.9%) | 41 (29.5%) | 0.01 |
| **Other biotherapy** | Yes | 5 (0.7%) | 2 (1.3%) | 6 (2%) | 2 (1.4%) | 0 (0%) | 0.28 |
| **Theophyllin** | Yes | 38 (5.5%) | 21 (13.7%) | 24 (8.1%) | 5 (3.5%) | 8 (5.8%) | <0.01 |
| **Physical activity** | Never | 189 (28,7%) | 64 (45,4%) | 111 (39,2%) | 38 (27,3%) | 28 (21,2%) | <0,0001 |
|  | Occasionally | 265 (40,2%) | 48 (34%) | 101 (35,7%) | 47 (33,8%) | 55 (41,7%) |  |
|  | Regularly | 147 (22,3%) | 25 (17,7%) | 59 (20,8%) | 44 (31,7%) | 38 (28,8%) |  |
|  | Frequent /competition | 58 (8,8%) | 4 (2,8%) | 12 (4,2%) | 10 (7,2%) | 11 (8,3%) |  |

S4 Figure : distribution of vems by cluster

S5 Figure : distribution of eosinophils by cluster

Table S6 : Blood count eosinophils according to OCS

| **Without OCS** | **Cluster 1**  **Early onset atopic  (n=613)** | | | **Cluster 2**  **Obese  (N=107)** | **Cluster 3**  **Late-onset**  **(N=235)** | **Cluster 4**  **Eosinophilic  (N=119)** | **Cluster 5**  **Aspirin-sensitivity  (N=108)** |  |
| --- | --- | --- | --- | --- | --- | --- | --- | --- |
| **Means**  **IC95%**  **Median**  **With OCS** | |  | 360.5  330.7-390.2  290  Cluster 1  (n=77) | 382.4  250.3-514.5  240.5  Cluster 2  (n=46) | 369.9  314.6-425.2  239  Cluster 3  (n=62) | 717.5  565.3-869.7  520  Cluster 4  (n=24) | 448.7  361.3-536  300  Cluster 5  (n=31) |  |
| **Means**  **IC95%**  **Median** | |  | 477.8  352.2-603.5  330 | 585.3  391.2-779.4  444 | 524.4  363-685.9  300 | 664.8  492.6-836.9  565 | 443.9  298.2-589.6  400 |  |
